# Cognitive Functioning in Youth with Anxiety Disorders: A Systematic Review

**DOI:** 10.1007/s10567-024-00480-9

**Published:** 2024-06-03

**Authors:** Jonathan C. Rabner, Julia S. Ney, Philip C. Kendall

**Affiliations:** 1 Department of Psychology and Neuroscience, Temple University, 1701 North 13th Street, Philadelphia, PA 19122 USA; 2 Behavioral Psychology Department, Kennedy Krieger Institute, Baltimore, MD USA; 3 The Johns Hopkins University School of Medicine, Baltimore, MD USA

**Keywords:** Anxiety, Children and adolescents, Language, Executive functioning, Memory, Attention

## Abstract

Anxiety disorders are disorders involving cognition. Research on cognition in youth with anxiety can focus on cognitive content (e.g., self-talk) as well cognitive functioning. The present review examines domains of cognitive functioning (i.e., episodic memory, language, attention, executive functioning, motor skills, and visual functioning) in youth diagnosed with an anxiety disorder. A database search of Embase, PsycINFO, and PubMed yielded 28 studies that met inclusion criteria of youth aged 17 years or younger, a sample diagnosed with a principal anxiety disorder and a comparison sample of controls, a comparison between those samples, and use of a behavioral measure of neuropsychological performance. Findings did not identify any cognitive functioning strengths for anxious youth. Deficits were found in two domains (i.e., receptive language and motor skills) whereas no deficits were found in attention, visuospatial skills and one domain of executive functioning (i.e., inhibition). Most domains had mixed findings. Additional analysis indicated that anxiety disorders in youth are not associated with diminished IQ. Directions for future research are identified including (a) the prioritization of studies with larger, representative samples (b) the role of cognitive functioning as a predictor of anxiety treatment outcome (c) the examination of the effect of treatment on cognitive performance, and (d) the course of anxiety and potential impairment in cognitive functioning.

## Introduction

Anxiety disorders are one of the most prevalent mental health disorders in children and adolescents (henceforth youth) with estimates ranging from 10 to 32% (Costello et al., 2003; Merikangas et al., 2010). Anxiety disorders in youth are associated with an array of adverse outcomes, including impaired social functioning (Seeley et al., 2011), impaired occupational and family functioning (Essau et al., 2014; Swan & Kendall, 2016), academic underachievement and dropout (Van Ameringen et al., 2003; Woodward & Fergusson, 2001), and decreased life satisfaction (Dooley et al., 2015). Anxiety disorders are often theorized as disorders of thoughts or cognitions (Ingram & Kendall, 1987), involving affective, physiological, and behavioral responses. Not surprisingly the first-line treatment for youth with anxiety disorders is cognitive-behavioral therapy (CBT; Higa-McMillan et al., 2016; Walter et al., 2020), which places a focus on identifying thoughts as well as learning skills to manage fear and prevent avoidance behaviors (e.g., cognitive restructuring, problem solving, exposure; Gosch et al., 2006).

Research on cognition in youth with anxiety often focuses on the content of thoughts or the internal monologue (often referred to as “self-talk”; Latinjak et al., 2023; Treadwell & Kendall, 1996), which often focuses on threat, failure, or hostility (Schniering & Rapee, 2002, 2004). Intolerance of uncertainty, where negative cognitions about the unknown trigger a fear response, is another common focus of cognitive content research (e.g., Cowie et al., 2018; Kendall et al., 2020). In addition to cognitive content, research can focus on cognitive functioning, such as executive functioning and memory. Intact cognitive functioning is inherent in the ability to engage in components of CBT. For example, cognitive flexibility is required for successful cognitive restructuring, with studies supporting this relationship in adults (Holder et al., 2021; Johnco et al., 2013). Additionally, as CBT employs a skills model where youth learn skills and later practice those skills in feared situations (i.e., exposure), the ability to encode, retain, and retrieve information presented both visually and verbally is critical. A decreased memory store or impaired ability to access information from memory may inhibit content mastery. Likewise, impaired retrieval of information may impact inhibitory learning during exposures, whose goal is to create new, nonthreatening connections with the feared stimuli or situation (Craske et al., 2008), if those new associations are unable to be retrieved. In an overall fashion, language and attention are key to engaging in any psychological therapy. Receptive language is required for youth to be able to comprehend the material and expressive language is required for youth to adequately report their thoughts and feelings. Youth are required to sustain attention in sessions, which typically last 45 min to one hour, and also shift or divide their attention as session demands change. Attentional issues (e.g., an anxiety and ADHD comorbidity rate of about 25%; Jarrett & Ollendick, 2008), are likely to impact youth’s ability to engage in and focus on activities.

Prior reviews on cognitive functioning in adults (Castaneda et al., 2008; Hedges et al., 2019) suggest that only trends, rather than conclusions, can be identified due to a dearth of studies. Impairment in executive functioning (e.g., inhibition, problem solving, cognitive flexibility) was found across diagnoses, however, findings were mixed with regard to attention [i.e., mixed findings for adults with social anxiety disorder (SAD) and no differences for generalized anxiety disorder (GAD)]. Similarly, impairment in visuospatial skills (i.e., deficit in adults with SAD but not GAD) and memory (i.e., visual memory deficit in adults with GAD, but mixed findings for verbal and visual memory in adults with SAD and panic disorder) also varied by diagnosis. No differences in language were reported (Castaneda et al., 2008; Hedges et al., 2019). Less is known about the pattern of results found in studies of cognitive functioning in youth with anxiety disorders.

The present review provides a comprehensive and systematic examination of domains of cognitive functioning (i.e., episodic memory, language, attention, executive functioning, motor skills, and visual functioning) in youth diagnosed with an anxiety disorder. This is the first review to examine potential strengths and weaknesses in cognitive functioning associated with anxiety disorders in youth. As such, the present review focuses on cognitive functioning rather than cognitive content or cognitive biases (e.g., memory, processing, attention) in youth with anxiety which are reviewed elsewhere (e.g., Dudeney et al., 2015). Through this aggregation of findings, we identify gaps in the literature and areas for future research, including domains that may serve as potential predictors or moderators of differential treatment outcome.

## Method

### Search Parameters

The literature search used Embase, PsycINFO, and PubMed databases including publications through December 2022. The following search of any aspect of an article (e.g., title, abstract, keyword) was conducted: “(anxiety disorder) AND (neuropsych* OR intelligence OR intellectual OR IQ OR memory OR language OR verbal OR visual OR spatial OR motor OR attention OR executive function* OR processing speed OR cognitive flexibility OR working memory OR inhibition OR planning OR problem solving OR shift*).” Study results were then filtered by age (i.e., 17 and under), language (i.e., English), and, when an option, publication type (i.e., journal article, conference presentation, or dissertation study) and subject (i.e., human studies).

### Inclusion/Exclusion Criteria and Eligibility

After duplicate studies were removed, the remaining 15,887 studies were subjected to the following inclusion and exclusion criteria. Inclusion criteria were: (1) majority of sample aged 17 years or younger; (2) at least one sample was diagnosed with a principal anxiety disorder – studies were permitted to focus on one disorder (e.g., GAD), multiple disorders, or collapse across all anxiety disorders into one anxiety disorder group; (3) a comparison sample of healthy controls, operationalized differently in each study but generally defined as a distinct group recruited to be without current anxiety or other diagnoses (henceforth referred to simply as “controls”); (4) a comparison between youth with an anxiety disorder and the controls; and (5) use of a behavioral measure of cognitive functioning (i.e., not a self-, parent-, or teacher-report questionnaire) to limit bias. Exclusion criteria were: (1) the study was focused on anxiety in youth with a medical disorder where the disorder may also impact cognitive functioning; and (2) the study solely included samples of youth with a different primary disorder (e.g., attention-deficit/hyperactivity disorder) or developmental disorder (e.g., autism spectrum disorder) and comorbid anxiety.

Due to changes in disorder classification from the 4th to 5th edition of the Diagnostic and Statistical Manual of Mental Disorders (American Psychiatric Association, 2013), studies with samples solely diagnosed with Obsessive–Compulsive Disorder or Posttraumatic Stress Disorder were not included. The inclusion criteria of a principal anxiety disorder also contributed to the exclusion of several studies (e.g., Emerson et al., 2005) that examined cognitive functioning in youth with both an anxiety and a depressive disorder or in mixed samples, given the known association between aspects of cognitive functioning and other diagnoses like depression (Goodall et al., 2018). Additionally, the inclusion criteria of a comparison sample of controls led to the exclusion of studies comparing youth with and without an anxiety disorder where the “no anxiety disorder” group contained youth with other diagnoses (e.g., Micco et al., 2009) to ensure that other diagnoses were not impacting results.

**Fig. 1:**
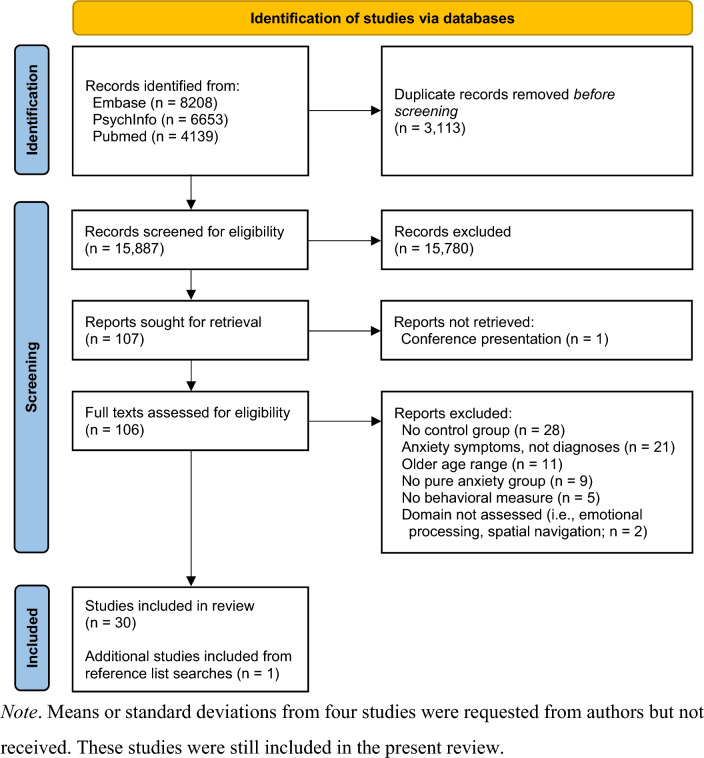
PRISMA flow diagram

Inclusion screening was conducted separately by the first and second authors with 99.2% agreement on the first stage of review (i.e., eligibility) and 100% agreement on the second stage of review (i.e., full text). Discrepancies were decided by consensus. As in Fig. 1 (the PRISMA flow diagram), an initial screen of study titles and abstracts resulted in 107 studies whose full texts were further screened for eligibility. An abstract for one conference presentation was deemed eligible for full review, however, additional requested information was unable to be provided by the authors. Following this screening, 30 studies were included. Reference lists of included studies were also reviewed and resulted in the inclusion of one additional study (i.e., Kristensen & Oerbeck, 2006).

### Data Extraction

Though not a statistical meta-analysis, to contextualize findings, sample size, age range, measure, and group means for all outcomes were extracted from studies following full-text screening by the first and second authors. 100% agreement on data extraction was found. Three studies were included without summary data because means and standard deviations were requested but not provided by the authors. When means and standard deviations were reported but pairwise comparative analysis was not conducted, independent sample t-tests were conducted using summary data comparing the anxiety group(s) to the controls. An alpha level of 0.05 was used unless a specific level was explicitly stated (e.g., Hybel et al., 2017 where a Bonferroni corrected p-value with seven tests equal to 0.007 was used). For all comparative analyses, effect sizes were calculated using Cohen’s d.

## Results

The results of studies are reviewed under separate headings consistent with the domains identified by the authors in the reported investigations. An integration of findings and conclusions are discussed in the subsequent section. Specific information on measures used, sample size, age range, group means, degree of significance, and effect size for all outcomes are reported in Tables 1, 2, 3, 4, 5, 6, 7, and 8.

**Table 1:** Episodic memory

Study	Samples (n)	Age range	Measure	Anxiety & CTRL (means)	Sig Diff?	Effect size (d)
*Visual memory*						
Jarros et al. (2017)	ANX (41 – split into 13 severe and 28 mild) CTRL (27)	10–17 years	ROCF (Delayed)	Severe ANX (16.1), CTRL (16.8)	No	0.12
				Mild ANX (18.8), CTRL (16.8)	No	0.29
Kim et al. (2019)	GAD (34) OCD (28) CTRL (65)	7–17 years	CANTAB (Pattern Recognition Memory—Number Correct)	GAD (21.47), CTRL (22.09)	No	0.24
Kristensen and Oerbeck (2006)	SM (32) CTRL (62)	6–17 years	Pointing to cubes on a tray in order task	SM (5.2), CTRL (5.2)	No	0.00
			BVRT (# correct)	SM (4.8) < CTRL (6.3)	**Yes**	**0.53**
Manassis et al. (2007a)	SM (44) ANX (28) CTRL (19)	6–10 years	Visual Patterns Test	SM (67.89) < CTRL (81.74)	**Yes**	**0.86**
				ANX (77.18), CTRL (81.74)	No	0.28
Toren et al. (2000)	ANX (19) CTRL (14)	6–18 years	ROCF (Immediate)	ANX (17.6), CTRL (18.7)	No	0.17
			ROCF (Delayed)	ANX (17.4), CTRL (18.3)	No	0.15
Vasa et al. (2007)	ANX (57) CTRL (103)	9–20 years	WRAML	ANX (8.39) < CTRL (9.75)	**Yes**	**0.62**
*Verbal memory*						
Günther et al. (2004)	ANX (34) DEP (31) CTRL (33)	6–17 years	RAVLT (Free Recall)	ANX (28.6), CTRL (31.5)	No	0.49
			RAVLT (Delayed Recall)	ANX (13.4), CTRL (13.8)	No	0.23
			RAVLT (Recognition)	ANX (14.6), CTRL (14.8)	No	0.26
Jarros et al. (2017)	ANX (41—split into 13 severe and 28 mild) CTRL (27)	10–17 years	WMS-III A + B (Immediate)	Severe ANX (21.2), CTRL (21.2)	No	0.00
				Mild ANX (23.4), CTRL (21.2)	No	0.35
			WMS-III A + B (Delayed)	Severe ANX (18.5), CTRL (18.3)	No	0.03
				Mild ANX (21.5), CTRL (18.3)	No	0.54
			RAVLT (1st list)	Severe ANX (4.8), CTRL (5.1)	No	0.17
				Mild ANX (5.1), CTRL (5.1)	No	0.00
			RAVLT (7th list)	Severe ANX (9.1), CTRL (10.0)	No	0.38
				Mild ANX (10.0), CTRL (10.0)	No	0.00
Kristensen and Oerbeck (2006)	SM (32) CTRL (62)	6–17 years	WISC-R (Digit Span)	SM (5.2) < CTRL (5.9)	**Yes**	**0.58**
Toren et al. (2000)	ANX (19) CTRL (14)	6–18 years	CVLT (Immediate Recall)	ANX (11.5), CTRL (12.9)	No	0.29
			CVLT (Delayed Recall)	ANX (11.6), CTRL (12.0)	No	0.13
			CVLT (Recognition)	ANX (13.1), CTRL (14.4)	No	0.68
Vasa et al. (2007)	ANX (57) CTRL (103)	9–20 years	WRAML	ANX (10.51), CTRL (10.90)	No	0.12
*Combined verbal memory, visual memory, and working memory*						
Toazza et al. (2014)	ANX (16) EXT (11) ANX + EXT (7) CTRL (23)	12–18 years	NEUPSILIN	ANX (58.8), CTRL (55.87)	No	0.31

Studies are presented in alphabetical order. Bold effect sizes indicate a significant difference. Sig Diff = significant difference

Samples: ANX = mixed anxiety; CTRL = healthy control; DEP = depressive disorder; EXT = externalizing disorders; GAD = generalized anxiety disorder; OCD = obsessive compulsive disorder; SM = selective mutism

Measures: BVRT = Benton Visual Retention Test; CANTAB = Cambridge Automated Neuropsychological Battery; CVLT = California Verbal Learning Test; RAVLT = Rey Auditory Verbal Learning Test; ROCF = Rey-Osterrieth Complex Figure Test; WISC = Wechsler Intelligence Scale for Children; WMS = Wechsler Memory Scale; WRAML = Wide Range Assessment of Memory and Learning

### Episodic Memory

Episodic memory (i.e., verbal and visual memory, but excluding working memory which is reviewed separately) was examined in eight studies (see Table 1; Günther et al., 2004; Jarros et al., 2017; Kim et al., 2019; Kristensen & Oerbeck, 2006; Manassis et al., 2007a; Toazza et al., 2014; Toren et al., 2000; Vasa et al., 2007). Lower visual memory scores but not verbal memory scores were found for youth, aged 9–20 years, with any anxiety disorder (*n* = 57) compared to controls (*n* = 103; Vasa et al., 2007). Of note, these findings did not differ when participants 18 years and older were excluded (Vasa et al., 2007). In another study, lower visual memory scores were found for youth, aged 6–10 years, with selective mutism (SM; *n* = 44) compared to youth with other anxiety disorders (*n* = 28) and controls (*n* = 19), which did not differ from each other (Manassis et al., 2007a). Finally, worse verbal memory performance was found in a study comparing youth, aged 6–17 years, with SM (*N* = 32) to 62 age-, gender-, location-, and socioeconomic status (SES)-matched controls (Kristensen & Oerbeck, 2006). Regarding visual memory, that same study found worse performance on one visual memory task (i.e., Benton Visual Retention Test; Benton Sivan, 1991) but not another task specifically designed for this study that involved pointing to an increasing span of cubes (range of 2–9 cubes) on a tray (Kristensen & Oerbeck, 2006). Significant differences were not found in the five remaining studies examining verbal memory (Günther et al., 2004), visual memory (Kim et al., 2019), and both verbal and non-verbal memory (Jarros et al., 2017; Toazza et al., 2014; Toren et al., 2000).

### Language

Language was examined in seven studies (see Table 2), six of which reported a deficit in language abilities (Davis III et al., 2008; Kristensen & Oerbeck, 2006; Manassis et al., 2007a; Mason, 2017; Milic et al., 2020; Nowakowski et al., 2009; Toazza et al., 2014). Four studies examined receptive vocabulary skills in youth. First, youth, aged 6–10 years with SM (*n* = 30), with a different anxiety disorder (*n* = 46), and controls (*n* = 27) were contrasted with an additional focus on sex differences (Nowakowski et al., 2009). Impairment in receptive language was found for both youth with SM as well as youth with a different anxiety disorder compared to controls for girls, however, no difference was found for boys (Nowakowski et al., 2009). Next, receptive vocabulary skills were examined in a sample of youth, aged 3–7 years, with SM (*n* = 23), SAD (*n* = 17), or controls (*n* = 15; Milic et al., 2020). Results indicated that youth with SM scored significantly lower on a receptive vocabulary task than controls. Youth with SAD did not differ from either group (Milic et al., 2020). Additionally, lower receptive vocabulary was found in a study comparing youth, aged 6–17 years, with SM (*N* = 32) to 62 age-, gender-, location-, and SES-matched controls (Kristensen & Oerbeck, 2006). Finally, in a study comparing youth, aged 6–10 years, with SM (*n* = 44), other anxiety disorders (*n* = 28), and controls (*n* = 19), youth with SM performed worse on two measures of receptive vocabulary than both youth with other anxiety disorder and controls, who did not differ from each other (Manassis et al., 2007a). The same pattern of differences was also found for another language metric, phonological awareness (Manassis et al., 2007a). No difference was found in one study that combined receptive and expressive language into one composite score (Davis III et al., 2008). Additionally, a combined measure of oral and written language was examined in one study (Toazza et al., 2014). No difference was found in combined oral and written language. Finally, pragmatic language skills, or the ability to interpret and use appropriate communication skills in different situations, was found to be lower in youth with anxiety (*n* = 18) compared to controls (*n* = 20; Mason, 2017).

**Table 2:** Language

Study	Samples (n)	Age range	Measure	Anxiety & CTRL (means)	Sig Diff?	Effect size (d)
*Receptive vocabulary*						
Kristensen and Oerbeck (2006)	SM (32) CTRL (62)	6–17 years	PPVT-3	SM (99.5) < CTRL (113.7)	**Yes**	**0.63**
Manassis et al. (2007a)	SM (44) ANX (28) CTRL (19)	6–10 years	PPVT-3	SM (97.35) < CTRL (110.95)	**Yes**	**0.96**
				ANX (111.00), CTRL (110.95)	No	0.00
			TROG	SM (89.86) < CTRL (102.26)	**Yes**	**0.70**
				ANX (100.86), CTRL (102.26)	No	0.09
Milic et al. (2020)	SM (25) Soc (17) CTRL (17)	3–7 years	PPVT-IV	SM (95.4) < CTRL (114.1); SOC (105.2)	**Yes**	**1.38**
Nowakowski et al. (2009)	SM (30–13 female) ANX (46–19 female) CTRL (27–14 female)	6–10 years	PPVT-3	Female: SM (105.92) < CTRL (116.07)	**Yes**	**1.11**
				Female: ANX (102.53) < CTRL (116.07)	**Yes**	**1.16**
				Male: SM (102.67), CTRL (108.58)	No	0.44
				Male: ANX (108.83), CTRL (108.58)	No	0.02
*Combined receptive and expressive language*						
Davis III et al. (2008)	Pure ANX: ANX (12) CTRL (17)	7–16 years	WIAT	ANX (108.7), CTRL (113.8)	No	0.32
*Combined oral and written language*						
Toazza et al. (2014)	ANX (16) EXT (11) ANX + EXT (7) CTRL (23)	12–18 years	NEUPSILIN	ANX (5.62), CTRL (5.35)	No	0.14
*Phonemic awareness*						
Manassis et al. (2007a)	SM (44) ANX (28) CTRL (19)	6–10 years	LACT (Total)	SM (66.08) < CTRL (79.56)	**Yes**	**0.63**
				ANX (80.11), CTRL (79.56)	No	0.03
*Pragmatic language*						
Mason (2017)	ANX (18) CTRL (20)	8–12 years	TOPL-2	ANX (105.78) < CTRL (114.80)	**Yes**	**0.88**

Studies are presented in alphabetical order. Bold effect sizes indicate a significant difference. Sig Diff = significant difference

Samples: ANX = mixed anxiety; CTRL = healthy control; EXT = externalizing disorders; SM = selective mutism; Soc = social anxiety disorder

Measures: LACT = Lindamood Auditory Conceptualization Test; NEUPSILIN = Brazilian Brief Neuropsychological Assessment Battery; PPVT = Peabody Picture Vocabulary Test; TOPL = Test of Pragmatic Language; TROG = Test of Reception Grammar; WIAT = Wechsler Individual Achievement Test

### Attention

Attention was examined in six studies (see Table 3; Baving et al., 2004; Günther et al., 2004; Jarros et al., 2017; Mogg et al., 2015; Toazza et al., 2014; Werry et al., 1987). Five studies reported no significant differences in attention (e.g., response time, errors) between youth with an anxiety disorder and controls (Baving et al., 2004; Günther et al., 2004; Jarros et al., 2017; Toazza et al., 2014; Werry et al., 1987). The other study examined specific attention networks, or components of attention (i.e., executive attention, orienting and alerting), in youth, aged 6–12 years, with anxiety (*N* = 67) and controls (*N* = 726; Mogg et al., 2015). No differences were found for any attention network when the anxiety group was examined together; however, when the anxiety group was split into youth with a specific phobia (*N* = 21) and youth with other anxiety disorders (*N* = 43), slower reaction times indicative of worse executive attention (i.e., the ability to resolve attentional conflicts) were found for youth with other anxiety diagnoses compared to both youth with a specific phobia and controls, who did not differ (Mogg et al., 2015).

**Table 3:** Attention

Study	Samples (n)	Age range	Measure	Anxiety & CTRL (means)	Sig Diff?	Effect size (d)
Baving et al. (2004)	ANX (16) CTRL (22)	11 years	CPT-AX (Hits)	ANX (39.1), CTRL (39.1)	No	0.00
			CPT-AX (Reaction Time)	ANX (438.8), CTRL (428.0)	No	0.19
			CPT-AX (Commission Errors)	ANX (0.9), CTRL (1.5)	No	0.46
Günther et al. (2004)	ANX (34) DEP (31) CTRL (33)	6–17 years	Simple RT task	ANX (250), CTRL (247)	No	0.07
			Sustained Attention Task (Tempo)	ANX (12.6), CTRL (11.7)	No	0.24
			Sustained Attention Task (misses)	ANX (19.4), CTRL (27.2)	No	0.43
			Sustained Attention Task (false alarms)	ANX (23.5), CTRL (19.1)	No	0.24
			Auditory-visual discrimination task (RT)	ANX (741), CTRL (711)	No	0.34
			Auditory-visual discrimination task (misses)	ANX (3.2), CTRL (3.9)	No	0.27
			Auditory-visual discrimination task (false alarms)	ANX (2.2), CTRL (2.9)	No	0.24
Jarros et al. (2017)	ANX (41 – split into 13 severe and 28 mild) CTRL (27)	10–17 years	D2	Severe ANX (154.4), CTRL (164.6)	No	0.11
				Mild ANX (178.5), CTRL (164.6)	No	0.16
Toazza et al. (2014)	ANX (16) EXT (11) ANX + EXT (7) CTRL (23)	12–18 years	NEUPSILIN	ANX (24.94), CTRL (25.09)	No	0.10
Werry et al. (1987)	ANX (21) ADHD (39) ADHD + CD/ODD (35) CTRL (21)^a^	5–13 years	CPT (Omission errors)	ANX (0.29), CTRL (0.67)	No	*
			CPT (Commission errors)	ANX (1.7), CTRL (1.3)	No	*
*Specific attention networks*						
Mogg et al. (2015)	ANX (67) later split into 21 with SP and 43 with other anx dxs ADHD (67) CTRL (726)	6–12 years	Executive Attention (ANT—RT)	ANX (91.7), CTRL (76.5)	No	0.20
				Other ANX (109.4) > CTRL (76.5)	**Yes**	**0.41**
			Orienting (ANT—RT)	ANX (23.7), CTRL (32.4)	No	0.13
			Alerting (ANT—RT)	ANX (62.1), CTRL (77.9)	No	0.20

Studies are presented in alphabetical order. Bold effect sizes indicate a significant difference. Sig Diff = significant difference

Samples: ADHD = attention-deficit/hyperactivity disorder (i.e., mixed or undefined types); ANX = mixed anxiety; CD/ODD = conduct disorder/oppositional defiant disorder CTRL = healthy control; DEP = depressive disorder; SP = specific phobia

Measures: ANT = Attention Network Task; CPT = Conners Continuous Performance Test; CPT-AX = Conners Continuous Performance Test-AX Version; RT = reaction time

^a^Different control sample sizes were used for different comparisons. The sample size listed was that used in comparisons with the ANX group

*Standard deviations were requested but not provided

### Executive Functioning

While organized into separate subdomains for the sake of clarity, it is worth noting that distilling measures of executive functioning into single components is not often simple. Findings from the following reviewed studies may have implications on other subdomains of executive functioning.

#### Working Memory

Nine studies examined differences in verbal and non-verbal (e.g., visual, spatial) working memory (see Table 4; Günther et al., 2004; Hybel et al., 2017; Jarros et al., 2017; John, 2005; Kim et al., 2019; Manassis et al., 2007a, 2007b; Mueller et al., 2015; Vance et al., 2013). Verbal working memory was examined in four studies. In a study comparing youth, aged 10–17 years, with anxiety to controls (*N* = 27), better performance on a measure of verbal working memory (i.e., Digit Span Backward; Wechsler, 2003) was found for youth with mild anxiety (*N* = 13) compared to both controls and youth with more severe anxiety (*N* = 28), who did not differ (Jarros et al., 2017). No significant difference was found on Digit Span Forward (Wechsler, 2003) in the same study (Jarros et al., 2017). Fewer correct responses on two complex verbal working memory tasks (i.e., CHIPASAT 2.0 s and 2.8 s; Johnson et al., 1988) were found in a study comparing youth, aged 7–12 years, with anxiety (*N* = 91) to controls (*N* = 34; John, 2005). Differences were not found with regard to errors on those tasks as well as on a simple verbal working memory task. No differences were found in the two remaining studies examining verbal working memory (Günther et al., 2004; Manassis et al., 2007a, 2007b).

**Table 4:** Working memory

Study	Samples (n)	Age range	Measure	Anxiety & CTRL (means)	Sig Diff?	Effect size (d)
*Verbal working memory*						
Günther et al. (2004)	ANX (34) DEP (31) CTRL (33)	6–17 years	RAVLT	ANX (6.6), CTRL (7.7)	No	0.46
Jarros et al. (2017)	ANX (41—split into 13 severe and 28 mild) CTRL (27)	10–17 years	WISC-IV (Digit Span Forward)	Severe ANX (7.1), CTRL (8.0)	No	0.44
				Mild ANX (8.1), CTRL (8.0)	No	0.05
			WISC-IV (Digit Span Backward)	Severe ANX (4.1), CTRL (3.7)	No	0.22
				Mild ANX (5.4) > CTRL (3.7)	**Yes**	**0.83**
John (2005)	ANX (91) CTRL (34)	7–12 years	WISC-3 (Digit Span Backward)	ANX (9.89), CTRL (10.59)	No	0.22
			CHIPASAT 2.0 s (correct)	ANX (24.09) < CTRL (29.25)	**Yes**	**0.59**
			CHIPASAT 2.0 s (commission errors)	ANX (2.98), CTRL (2.17)	No	0.29
			CHIPASAT 2.0 s (omission errors)	ANX (32.95), CTRL (28.58)	No	0.47
			CHIPASAT 2.8 s (correct)	ANX (29.21) < CTRL (35.16)	**Yes**	**0.57**
			CHIPASAT 2.8 s (commission errors)	ANX (4.64), CTRL (3.44)	No	0.25
			CHIPASAT 2.8 s (omission errors)	ANX (26.18), CTRL (21.40)	No	0.45
Manassis et al. (2007b)	ANX (52) ANX + ADHD (35) ADHD (21) CTRL (35)	8–12 years	WISC-III (Digit Span Forward)	ANX (10.81), CTRL (10.63)	No	0.06
			WISC-III (Digit Span Backward)	ANX (10.67), CTRL (10.4)	No	0.09
			CHIPASAT 2.8 s (correct)	ANX (31.49), CTRL (34.88)	No	0.31
			CHIPASAT 2.0 s (correct)	ANX (25.51), CTRL (29.36)	No	0.42
*Non-verbal working memory*						
Hybel et al. (2017)	ANX (38) OCD (50) CTRL (50)	7–17 years	CANTAB (SWM—errors)	ANX (32.21) > CTRL (21.88)	**Yes**	**0.59**
			CANTAB (Spatial Span—length)	ANX (5.53) < CTRL (6.39)	**Yes**	**0.72**
John, 2005	ANX (91) CTRL (34)	7–12 years	Finger Windows (Backward)	ANX (9.88), CTRL (11.31)	No	0.34
			SOPT (6-item)	ANX (0.70), CTRL (0.75)	No	0.06
			SOPT (8-item)	ANX (1.15), CTRL (1.09)	No	0.06
			SOPT (10-item)	ANX (1.26), CTRL (1.12)	No	0.14
			SOPT (12-item)	ANX (2.10), CTRL (2.12)	No	0.01
Kim et al. (2019)	GAD (34) OCD (28) CTRL (65)	7–17 years	Spatial Span (length)	GAD (6.21), CTRL (6.58)	No	0.26
			Spatial Span (errors)	GAD (14.15), CTRL (14.75)	No	0.08
Manassis et al. (2007a)	SM (44) ANX (28) CTRL (19)	6–10 years	Corsi Blocks (Forward)	SM (8.86) < CTRL (11.78)	**Yes**	**1.01**
				ANX (10.79), CTRL (11.78)	No	0.31
			Corsi Blocks (Backward)	SM (9.34) < CTRL (11.89)	**Yes**	**0.84**
				ANX (9.93), CTRL (11.89)	No	0.72
			Finger Windows (Forward)	SM (10.00), CTRL (11.58)	No	0.48
				ANX (9.61), CTRL (11.58)	No	0.57
			Finger Windows (Backward)	SM (7.16), CTRL (10.26)	No	0.77
				ANX (10.71), CTRL (10.26)	No	0.11
Manassis et al. (2007b)	ANX (52) ANX + ADHD (35) ADHD (21) CTRL (35)	8–12 years	Finger Windows Forward	ANX (8.91), CTRL (9.39)	No	0.15
			Finger Windows Backward	ANX (10.13), CTRL (11.33)	No	0.28
Mueller et al. (2015)	ANX (33) CTRL (22)	8–12 years	Saccadic Working Memory Task	*	No	*
Vance et al. (2013)	ANX (69) CTRL (116) ADHD (163) ANX + ADHD (116)	6–16 years	CANTAB (Spatial Span—errors)	ANX (37.88) > CTRL (29.84)	**Yes**	**0.49**
			CANTAB (Spatial Span—strategy)	ANX (34.57), CTRL (33.19)	No	0.24
			CANTAB (Spatial Span—span)	ANX (5.95) < CTRL (6.45)	**Yes**	**0.35**

Studies are presented in alphabetical order. Bold effect sizes indicate a significant difference. Sig Diff = significant difference

Samples: ADHD = attention-deficit/hyperactivity disorder (i.e., mixed or undefined types); ANX = mixed anxiety; CTRL = healthy control; DEP = depressive disorder; GAD = generalized anxiety disorder; OCD = obsessive compulsive disorder; SM = selective mutism

Measures: CANTAB = Cambridge Automated Neuropsychological Battery; CHIPASAT = Children’s Paced Auditory Serial Addition Task; RAVLT = Rey Auditory Verbal Learning Test; SOPT = Self-Ordered Pointing Task; SWM = Spatial Working Memory; WISC = Wechsler Intelligence Scale for Children

*Individual means were requested but not provided

Non-verbal (e.g., visual, spatial) working memory was examined in seven studies. Worse spatial working memory performance (i.e., number of errors and longest span but not strategy) was found in a study comparing youth, aged 6–16 years, with anxiety (*N* = 69) to 116 age- and gender-matched controls (Vance et al., 2013). Worse spatial working memory was also found on two measures (i.e., errors and span length) in a second study of youth, aged 7–17 years, with anxiety disorders (*n* = 38) compared to controls (*n* = 50; Hybel et al., 2017). In another study, youth, aged 6–10 years, with SM (*n* = 44), other anxiety disorders (*n* = 28), and controls (*n* = 19) completed several measures of spatial working memory (Manassis et al., 2007a). Youth with SM performed worse that controls on some (i.e., Corsi Blocks Forward and Backward; Wechsler, 1991) but not all (i.e., Finger Windows Forward and Backward; Sheslow & Adams, 1990) spatial working memory tasks. Furthermore, youth with SM performed worse that youth with other anxiety disorders on the Corsi Blocks Forward task (Wechsler, 1991), however, youth with other anxiety disorders did not differ from controls (Manassis et al., 2007a). No differences were found in the four remaining studies examining visuospatial working memory (John, 2005; Kim et al., 2019; Manassis et al., 2007b; Mueller et al., 2015).

#### Cognitive Flexibility

Four studies examined differences in cognitive flexibility (see Table 5; Hybel et al., 2017; Jarros et al., 2017; Kim et al., 2019; Toren et al., 2000). Lower cognitive flexibility was found in youth with an anxiety disorder (*n* = 19; aged 6–18 years) compared to 14 age-matched controls on several metrics (Toren et al., 2000). Youth with anxiety made more total errors and perseverate errors and also answered more questions incorrectly after negative feedback than controls, however, there was no difference between the two groups with respect to correct responses after positive feedback (Toren et al., 2000). In another study, youth with GAD (*n* = 34; aged 7–17 years) demonstrated worse cognitive flexibility compared to 65 controls with regard to simple reversal errors (i.e., errors when only one dimension is present in stimuli) but not total reversal errors (i.e., combined errors when one or both dimensions are present; Kim et al., 2019). No differences were found in the two remaining studies examining cognitive flexibility (Hybel et al., 2017; Jarros et al., 2017).

**Table 5:** Executive functioning (i.e., cognitive flexibility, processing speed, inhibition, planning, cognitive control, and problem solving)

Study	Samples (*n*)	Age range	Measure	Anxiety & CTRL (means)	Sig Diff	Effect size (d)
*Cognitive flexibility*						
Hybel et al. (2017)	ANX (38) OCD (50) CTRL (50)	7–17 years	CANTAB (Intra/Extra Dimensional Set Shift)	ANX (35.74), CTRL (26.08)	No	0.37
Jarros et al. (2017)	ANX (41 – split into 13 severe and 28 mild) CTRL (27)	10–17 years	WCST (Total correct)	Severe ANX (77.7), CTRL (81.1)	No	0.27
				Mild ANX (78.6), CTRL (81.1)	No	0.20
			WCST (Perseverative errors)	Severe ANX (20.7), CTRL (17.3)	No	0.41
				Mild ANX (18.3), CTRL (17.3)	No	0.12
Kim et al. (2019)	GAD (34) OCD (28) CTRL (65)	7–17 years	IDED (simple reversal errors)	GAD (2.29) > CTRL (1.43)	**Yes**	**0.51**
			IDED (total reversal errors)	GAD (10.18), CTRL (7.26)	No	0.31
Toren et al. (2000)	ANX (19) CTRL (14)	6–18 years	WCST (Total errors)	ANX (31.6) > CTRL (19.8)	**Yes**	**0.93**
			WCST (Perseverative responses)	ANX (15.1) > CTRL (9.6)	**Yes**	**1.05**
			WCST (Correct after positive feedback)	ANX (75.6), CTRL (79.0)	No	0.31
			WCST (Incorrect after negative feedback)	ANX (31.6) > CTRL (21.2)	**Yes**	**0.78**
*Processing speed/efficiency*						
John (2005)	ANX (91) CTRL (34)	7–12 years	CHIPASAT 2.0 s (consecutive correct)	ANX (1.72), CTRL (1.85)	No	0.12
			CHIPASAT 2.0 s (consecutive omission errors)	ANX (2.41) > CTRL (1.78)	**Yes**	**0.56**
			CHIPASAT 2.8 s (consecutive correct)	ANX (2.21), CTRL (2.57)	No	0.20
			CHIPASAT 2.8 s (consecutive omission errors)	ANX (1.86), CTRL (1.42)	No	0.49
Kim et al. (2019)	GAD (34) OCD (28) CTRL (65)	7–17 years	CANTAB (Pattern Recognition Memory—Latency)	GAD (2495.98) > CTRL (2052.04)	**Yes**	**0.72**
*Inhibition/initiation*						
Günther et al. (2004)	ANX (34) DEP (31) CTRL (33)	6–17 years	Go/No-Go Paradigm (RT)	ANX (442), CTRL (389)	No	0.76
			Go/No-Go Paradigm (misses)	ANX (0.5), CTRL (0.8)	No	0.29
			Go/No-Go Paradigm (false alarms)	ANX (2.9), CTRL (2.2)	No	0.32
Hybel et al. (2017)	ANX (38) OCD (50) CTRL (50)	7–17 years	CANTAB (Stop Signal Task)	ANX (208.44), CTRL (181.23)	No	0.43
			Flanker Task	ANX (72.83), CTRL (75.93)	No	0.17
Jarros et al. (2017)	ANX (41 – split into 13 severe and 28 mild) CTRL (27)	10–17 years	Go/No-Go Paradigm (total hits)	Severe ANX (80.5), CTRL (82.1)	No	0.23
				Mild ANX (80.3), CTRL (82.1)	No	0.28
Korenblum et al. (2007)	ANX (21)—10 w/out subthreshold ADHD ADHD (78) ANX + ADHD (38) CTRL (40)	6–14 years	SS Paradigm (RT)	ANX (314) > CTRL (238)	**Yes**	**0.61**
				ANX w/out subthreshold ADHD (256.70), CTRL (238)	No	0.19
Manassis et al. (2000)	ANX (14) ADHD (14) ANX + ADHD (16) CTRL (13)	8–12 years	SS Task (Go RT)	ANX (567.69), CTRL (567.45)	No	0.00
			SS Task (SS RT)	ANX (237.33), CTRL (236.98)	No	0.00
*Planning*						
Hybel et al. (2017)	ANX (38) OCD (50) CTRL (50)	7–17 years	SOC	ANX (7.73), CTRL (8.62)	No	0.45
Kim et al. (2019)	GAD (34) OCD (28) CTRL (65)	7–17 years	SOC (2 moves)	GAD (2.06), CTRL (2.05)	No	0.04
			SOC (3 moves)	GAD (3.29), CTRL (3.25)	No	0.09
			SOC (4 moves)	GAD (5.63), CTRL (5.43)	No	0.20
			SOC (5 moves)	GAD (7.53), CTRL (7.03)	No	0.36
Rodrigues et al. (2019)	ANX (37) CTRL (34)	7–17 years	TOH (3 pieces—time)	ANX (38) > CTRL (21.3)	**Yes**	**0.68**
			TOH (3 pieces—moves)	ANX (13.0) > CTRL (8.9)	**Yes**	**0.84**
			TOH (3 pieces—errors)	ANX (2.5), CTRL (1.5)	No	0.37
			TOH (4 pieces—time)	ANX (83.4) > CTRL (57.4)	**Yes**	**0.85**
			TOH (4 pieces—moves)	ANX (31.6), CTRL (24.9)	No	0.73
			TOH (4 pieces—errors)	ANX (5.4) > CTRL (2.0)	**Yes**	**0.80**
*Cognitive control*						
Cardinale et al. (2019)	ANX (35) CRTL (22)	M = 12.8, SD = 3.11 years ^a^	Antisaccade task (% correct prosaccade)	*	No	*
			Antisaccade task (% correct antisaccade)	*	No	*
			Antisaccade task (prosaccade errors)	*	No	*
			Antisaccade task (antisaccade errors)	*	No	*
			Antisaccade task (prosaccade latency)	*	No	*
			Antisaccade task (antisaccade latency)	*	**Yes**	*
Hardin et al. (2007)	ANX (16) DEP (11) CTRL (30)	9–17 years	Antisaccade task (accuracy direction errors)	ANX (37.38), CTRL (29.24)	No	0.40
			Antisaccade task (accuracy correct)	ANX (45.46), CTRL (52.94)	No	0.32
Jazbec et al. (2005)	ANX (11) DEP (12) CRTL (28)	9–17 years	Antisaccade task (# incorrect)	ANX (9.36), CTRL (8.89)	No	0.35
			Antisaccade task (latency)	ANX (191.41), CTRL (208.21)	**Yes**	**1.92**
*Problem solving*						
Toazza et al. (2014)	ANX (16) EXT (11) ANX + EXT (7) CTRL (23)	12–18 years	NEUPSILIN	ANX (1.56), CTRL (1.57)	No	0.02
*Verbal fluency*						
Toazza et al. (2014)	ANX (16) EXT (11) ANX + EXT (7) CTRL (23)	12–18 years	NEUPSILIN	ANX (8.5) < CTRL (12.09)	**Yes**	**1.25**
*Multiple aspects of executive functioning (i.e., cognitive flexibility, processing speed, visual scanning)*						
Hybel et al. (2017)	ANX (38) OCD (50) CTRL (50)	7–17 years	TMT B (time)	ANX (47.14), CTRL (37.28)	No	0.51
Jarros et al. (2017)	ANX (41 – split into 13 severe and 28 mild) CTRL (27)	10–17 years	TMT A (time)	Severe ANX (72.4), CTRL (66.5)	No	0.27
				Mild ANX (61.1), CTRL (66.5)	No	0.27
			TMT A (errors)	Severe ANX (0.1), CTRL (0.1)	No	0.00
				Mild ANX (0.1), CTRL (0.1)	No	0.00
			TMT B (time)	Severe ANX (142.9), CTRL (132.5)	No	0.21
				Mild ANX (125.4), CTRL (132.5)	No	0.15
			TMT B (errors)	Severe ANX (0.3), CTRL (0.2)	No	0.08
				Mild ANX (0.1), CTRL (0.2)	No	0.08

Studies are presented in alphabetical order. Bold effect sizes indicate a significant difference. Sig Diff = significant difference; RT = reaction time

Samples: ADHD = attention-deficit/hyperactivity disorder (i.e., mixed or undefined types); ANX = mixed anxiety; CTRL = healthy control; DEP = depressive disorder; EXT = externalizing disorders; GAD = generalized anxiety disorder; OCD = obsessive compulsive disorder

Measures: CHIPASAT = Children’s Paced Auditory Serial Addition Task; NEUPSILIN = Brazilian Brief Neuropsychological Assessment Battery; ROCF = Rey-Osterrieth Complex Figure Test; SOC = Stockings of Cambridge; SS = Stop-signal; TMT = Trail Making Test; TOH = Tower of Hanoi; WCST = Wisconsin Card Sorting Task

^a^Age range not provided

*Individual means were requested but not provided

#### Processing Speed/Efficiency

Processing speed and processing efficiency were each examined in one study (see Table 5; John, 2005; Kim et al., 2019). Slower visual processing was found in youth, aged 7–17 years, with a diagnosis of GAD (*n* = 34) compared to controls (*n* = 65; Kim et al., 2019). Processing efficiency was operationalized as consecutive correct responses and consecutive omission errors in a study comparing youth, aged 7–12 years, with anxiety (*N* = 91) to controls (*N* = 34; John, 2005). Consecutive omission errors were greater in youth with anxiety than controls on the CHIPASAT (Johnson et al., 1988) 2.0 s task but not the 2.8 s task. Additionally, the two groups did not differ with respect to consecutive correct responses (John, 2005).

#### Inhibition/Initiation

Five studies examined differences in inhibition and/or initiation (see Table 5; Günther et al., 2004; Hybel et al., 2017; Jarros et al., 2017; Korenblum et al., 2007; Manassis et al., 2000). Slower reaction times on an inhibition task were found in youth with an anxiety disorder (*n* = 21; aged 6–14 years) compared to 40 controls, however, when 11 youth with subthreshold ADHD were excluded from the analysis, there was no difference between the remaining 10 anxious youth and the controls (Korenblum et al., 2007). No differences were found in the remaining four studies (Günther et al., 2004; Hybel et al., 2017; Jarros et al., 2017; Manassis et al., 2000).

#### Planning

Three studies examined differences in planning ability or efficiency (see Table 5; Hybel et al., 2017; Kim et al., 2019; Rodrigues et al., 2019), with one finding significant differences (Rodrigues et al., 2019). In that study, youth, aged 10–17 years, with an anxiety disorder (*n* = 37) were compared to controls (*n* = 34) utilizing the Tower of Hanoi task (Humes et al., 1997). Youth with anxiety required more time and more moves on the 3-disk task and required more time and made more error in the 4-disk when compared to their non-anxious counterparts (Rodrigues et al., 2019).

#### Cognitive Control

Three studies examined differences in cognitive control (i.e., the ability to regulate cognitive processes; see Table 5). No differences were found on most metrics (e.g., errors, accuracy) in one study comparing youth with anxiety (*n* = 35; M_age_ = 12.8 years) to controls (*n* = 22), with the exception of longer antisaccade (i.e., eye movement away from a stimulus) latency, but not prosaccade (i.e., eye movement towards a stimulus) latency (Cardinale et al., 2019). Conversely, shorter latency was found on incorrect trials of an antisaccade task for youth, aged 9–17 years, with an anxiety disorder (*n* = 11) compared to controls (*n* = 28), through no differences were found in the number of incorrect trials (Jazbec et al., 2005). No differences were found in the remaining study (Hardin et al., 2007).

#### Problem Solving

Problem solving was examined in one study which did not find a significant difference between youth with anxiety and controls (see Table 5; Toazza et al., 2014).

#### Verbal Fluency

Verbal fluency was examined in one study (Toazza et al., 2014). Worse verbal fluency was found for youth, aged 12–18 years, with anxiety (*n* = 16) compared to 23 controls (Toazza et al., 2014).

#### Multiple Aspects of Executive Functioning

Two studies used the Trail Making Test (Trail A and B (Reitan & Wolfson, 1993) for Jarros and colleagues (2017) and only Trail B for Hybel and colleagues (2017) to examine differences in multiple aspects of executive functioning (i.e., cognitive flexibility, processing speed, visual scanning). Neither study reported significant differences (see Table 5; Hybel et al., 2017; Jarros et al., 2017).

### Motor Skills

Motor functioning, including fine and gross motor impairment as well as motor coordination and motor praxis, was examined in six studies (see Table 6; Ekornås et al., 2010; Kristensen & Torgersen, 2008; Skirbekk et al., 2012; Toazza et al., 2014; Vance et al., 2006; Werry et al., 1987). Three studies examined fine motor skills in youth. Worse fine motor skills were found for youth, aged 6–12 years, with any anxiety disorder (*n* = 25) compared to youth (*n* = 20) on one measure (i.e., Scored Developmental Neurological Examination – Mirror Movements; (Kakebeeke et al., 1993), but not another (i.e., Scored Developmental Neurological Examination – Smoothness/accuracy; Vance et al., 2006). Significantly worse fine motor skills were found in another study comparing youth, aged 7–13 years, with anxiety disorders (*n* = 41) to controls (*n* = 36; Skirbekk et al., 2012), however, no differences were found using the same measure [i.e., Movement Assessment Battery for Children (MABC Henderson et al., 2007) – Manual Dexterity] in a different study (Kristensen & Torgersen, 2008). These same two studies also examined gross motor impairment and similarly found contrasting results. In a study comparing youth, aged 11–12 years, with SAD (*n* = 29) to controls (*n* = 48), gross motor impairment was found for youth with anxiety on the MABC – Balance subtest but not the MABC – Ball Skills subtest (Kristensen & Torgersen, 2008). Skirbekk and colleagues (2012) found the opposite findings of significant differences on the MABC – Ball Skills subtest but not the MABC – Balance subtest. Finally, when assessment of fine and gross motor skills were combined to yield a total motor impairment score, both studies found significantly greater impairment in youth with an anxiety disorder than in controls (Kristensen & Torgersen, 2008; Skirbekk et al., 2012). Similar results of combined fine and gross motor impairment for youth with an anxiety disorder (*n* = 27) were found in a third study that compared them to controls, aged 8–11 years, matched according to age, gender, and full-scale IQ (*n* = 27; Ekornås et al., 2010).

**Table 6:** Motor skills

Study	Samples (n)	Age range	Measure	Anxiety & CTRL (means)	Sig Diff?	Effect size (d)
*Fine motor impairment*						
Kristensen and Togerson (2008)	Soc (29) ADHD (23) Soc + ADHD (6) Other dx (44) CTRL (48)	11–12 years	MABC (Manual Dexterity)	Soc (6.9), CTRL (5.8)	No	.31
Skirbekk et al. (2012)	ANX (41) ADHD (39) ANX + ADHD (25) CTRL (36)	7–13 years	MABC (Manual Dexterity)	ANX (6.7) > CTRL (2.8)	**Yes**	**1.06**
Vance et al. (2006)	ANX (25) CTRL (20) ADHD-C (37) DD (37)	6–12 years	SDNE (smoothness / accuracy)	ANX (0.88), CTRL (0.56)	No	0.65
			SDNE (mirror movements)	ANX (1.21) > CTRL (0.25)	**Yes**	**1.71**
*Gross motor impairment*						
Kristensen and Togerson (2008)	Soc (29) ADHD (23) Soc + ADHD (6) Other dx (44) CTRL (48)	11–12 years	MABC (Ball Skills)	Soc (2.5), CTRL (1.3)	No	0.49
			MABC (Balance)	Soc (4.9) > CTRL (1.3)	**Yes**	**1.28**
Skirbekk et al. (2012)	ANX (41) ADHD (39) ANX + ADHD (25) CTRL (36)	7–13 years	MABC (Ball Skills)	ANX (2.2) > CTRL (1.2)	**Yes**	**0.51**
			MABC (Balance)	ANX (2.7), CTRL (1.4)	No	0.58
*Combined fine and fine gross impairment*						
Ekornås et al. (2010)	ANX (27) CTRL (27)	8–11 years	MABC	ANX (13.41) > CTRL (7.74)	**Yes**	**0.80**
Kristensen and Togerson (2008)	Soc (29) ADHD (23) Soc + ADHD (6) Other dx (44) CTRL (48)	11–12 years	MABC	Soc (14.3) > CTRL (8.4)	**Yes**	**0.92**
Skirbekk et al., 2012	ANX (41) ADHD (39) ANX + ADHD (25) CTRL (36)	7–13 years	MABC	ANX (11.7) > CTRL (5.4)	**Yes**	**1.15**
*Motor Coordination*						
Vance et al. (2006)	ANX (25) CTRL (20) ADHD-C (37) DD (37)	6–12 years	SDNE (choreoathetoid movements)	ANX (1.23), CTRL (0.75)	No	0.81
			SDNE (conjugate eye gaze)	ANX (0.67), CTRL (0.25)	No	0.68
Werry et al. (1987)	ANX (21) ADHD (39) ADHD + CD/ODD (35) CTRL (21)^a^	5–13 years	Mazes (Time)	ANX (3.0), CTRL (2.1)	No	*
			Mazes (Contacts)	ANX (25.8), CTRL (19.3)	No	*
			Holes (Time)	ANX (12.2), CTRL (12.5)	No	*
			Holes (Contacts)	ANX (114), CTRL (126)	No	*
			Pursuit Rotor (Time)	ANX (33.8), CTRL (38.3)	No	*
			Pursuit Rotor (Contacts)	ANX (53.3), CTRL (53.6)	No	*
*Motor praxis*						
Toazza et al. (2014)	ANX (16) EXT (11) ANX + EXT (7) CTRL (23)	12–18 years	NEUPSILIN	ANX (15.69), CTRL (15.91)	No	0.09

Studies are presented in alphabetical order. Bold effect sizes indicate a significant difference. Sig Diff = significant difference

Samples: ADHD = attention-deficit/hyperactivity disorder (i.e., mixed or undefined types); ADHD-C = attention-deficit/hyperactivity disorder – combined; ANX = mixed anxiety; CD/ODD = conduct disorder/oppositional defiant disorder; CTRL = healthy control; DD = dysthymic disorder; EXT = externalizing disorders; Soc = social anxiety disorder

Measures: MABC = Movement Assessment Battery for Children; NEUPSILIN = Brazilian Brief Neuropsychological Assessment Battery; SDNE = Scored Developmental Neurological Examination

^a^Different control sample sizes were used for different comparisons. The sample size listed was that used in comparisons with the ANX group

*Standard deviations were requested but not provided

Regarding motor coordination, no significant differences were found in both studies across multiple measures (Vance et al., 2006; Werry et al., 1987). Likewise, significant differences were not found in the study examining motor praxis (Toazza et al., 2014).

### Visual Functioning

Two studies examined differences in visuospatial skills (Jarros et al., 2017; Toren et al., 2000) and one study each examined differences in visual scanning (Lubow et al., 2000) and visual perception (Toazza et al., 2014). No study reported significant differences (see Table 7).

**Table 7:** Visual functioning (i.e., visuospatial skills, visual scanning, and visual perception)

Study	Samples (n)	Age range	Measure	Anxiety & CTRL (Means)	Sig Diff?	Effect Size (d)
*Visuospatial skills*						
Jarros et al. (2017)	ANX (41—split into 13 severe and 28 mild) CTRL (27)	10–17 years	ROCF (Copy)	Severe ANX (30.6), CTRL (27.5)	No	0.70
				Mild ANX (30.0), CTRL (27.5)	No	0.53
Toren et al. (2000)	ANX (19) CTRL (14)	6–18 years	ROCF (Copy)	ANX (30.4), CTRL (32.2)	No	0.72
*Visual scanning*						
Lubow et al. (2000)	ANX (23) CTRL (23)	6–17 years	Visual Search Task	*	No	*
*Visual perception*						
Toazza et al. (2014)	ANX (16) EXT (11) ANX + EXT (7) CTRL (23)	12–18 years	NEUPSILIN	ANX (1.19), CTRL (1.87)	No	0.48

Studies are presented in alphabetical order. Bold effect sizes indicate a significant difference. Sig Diff = significant difference

Samples: ANX = mixed anxiety; CTRL = healthy control; EXT = externalizing disorders; GAD = generalized anxiety disorder; OCD = obsessive compulsive disorder

Measures: CANTAB = Cambridge Automated Neuropsychological Battery; NEUPSILIN = Brazilian Brief Neuropsychological Assessment Battery; ROCF = Rey-Osterrieth Complex Figure Test

*Individual means were requested but not provided

### IQ

Included studies were also examined for comparisons of IQ between youth with anxiety disorders and healthy controls (see Table 8). Of the 31 included studies, 22 studies contained this comparison (Günther et al., 2004; Hardin et al., 2007; Hybel et al., 2017; Jarros et al., 2017; John, 2005; Kim et al., 2019; Korenblum et al., 2007; Kristensen & Oerbeck, 2006; Kristensen & Torgersen, 2008; Manassis et al., 2007a, 2007b; Manassis et al., 2007a, 2007b; Mogg et al., 2015; Mueller et al., 2015; Rodrigues et al., 2019; Skirbekk et al., 2012; Toazza et al., 2014; Toren et al., 2000; Vance et al., 2006, 2013; Vasa et al., 2007). Findings were mixed, with significant differences found in only seven of those studies. First, lower full scale IQ and verbal IQ but not performance IQ was found in youth, aged 6–16 years, with a diagnosis of SAD (*N* = 29) compared to 48 controls (Kristensen & Torgersen, 2008). Conversely, lower full scale IQ and performance IQ but not verbal IQ was found in youth, aged 6–16 years, with anxiety compared to controls (Davis III et al., 2008). Next, full scale IQ, performance IQ, and visual IQ were all significantly lower in a study comparing youth, aged 6–16 years, with anxiety (*N* = 69) to 116 controls (Vance et al., 2013). Additionally, lower performance IQ was found in one study comparing youth, aged 6–17 years, with SM (*N* = 32) to 62 age-, gender-, location-, and SES-matched controls (Kristensen & Oerbeck, 2006). Lastly, lower IQ was found in three studies comparing youth with an anxiety disorder to controls (Korenblum et al., 2007; Skirbekk et al., 2012; Vasa et al., 2007). Of note, in each of the five studies, all IQ scores still fell within the average range, indicating that these differences do not indicate diminished IQ.

**Table 8:** IQ

Study	Samples (n)	Age range	Measure	Anxiety & CTRL (means)	Sig Diff?	Effect size (d)
Cardinale et al. (2019)	ANX (35) CRTL (22)	M = 12.8, SD = 3.11 years ^a^	IQ (Measure not specified)	ANX (109.56), CTRL (109.94)	No	0.03
Davis III et al. (2008)	Pure ANX: ANX (12) CTRL (17)	7–16 years	FSIQ (WISC-3)	ANX (94.8) < CTRL (109.5)	**Yes**	**0.84**
			VIQ (WISC-3)	ANX (98.7), CTRL (108.8)	No	0.54
			PIQ (WISC-3)	ANX (90.4) < CTRL (108.5)	**Yes**	**1.03**
Günther et al. (2004)	ANX (34) DEP (31) CTRL (33)	6–17 years	FSIQ (WISC-3)	ANX (102), CTRL (107)	No	0.45
Hardin et al. (2007)	ANX (16) DEP (11) CTRL (30)	9–17 years	IQ (Measure not specified)	ANX (109.94), CTRL (110.30)	No	0.02
Hybel et al. (2017)	ANX (38) OCD (50) CTRL (50)	7–17 years	FSIQ (RIST)	ANX (102.55), CTRL (104.40)	No	0.25
			Verbal IQ (RIST)	ANX (103.48), CTRL (102.55)	No	0.12
			Non-verbal IQ (RIST)	ANX (101.50), CTRL (105.58)	No	0.40
Jarros et al. (2017)	ANX (41 – split into 13 severe and 28 mild) CTRL (27)	10–17 years	IQ (WASI)	Severe ANX (100.8), CTRL (118.7)	No	0.77
				Mild ANX (112.6), CTRL (118.7)	No	0.22
John (2005)	ANX (91) CTRL (34)	7–12 years	FSIQ (WISC-3)	ANX (105.72), CTRL (110.61)	No	0.38
Kim et al. (2019)	GAD (34) OCD (28) CTRL (65)	7–17 years	IQ (WASI)	GAD (110.2), CTRL (111.5)	No	0.11
Korenblum et al. (2007)	ANX (21)—10 w/out subthreshold ADHD ADHD (78) ANX + ADHD (38) CTRL (40)	6–14 years	IQ (WISC-3)	ANX (104.1) < CTRL (118.2)	**Yes**	**1.22**
Kristensen and Oerbeck (2006)	SM (32) CTRL (62)	6–17 years	PIQ (WISC-R or WPPSI)	SM (96.5) < CTRL (108.2)	**Yes**	**0.73**
Kristensen and Togerson (2008)	Soc (29) ADHD (23) Soc + ADHD (6) Other dx (44) CTRL (48)	11–12 years	FSIQ (WASI)	Soc (98.8) < CTRL (104.9)	**Yes**	**0.46**
			VIQ (WASI)	Soc (92.1) < CTRL (99.8)	**Yes**	**0.64**
			PIQ (WASI)	Soc (106.6), CTRL (109.3)	No	0.18
Manassis et al. (2007a)	SM (44) ANX (28) CTRL (19)	6–10 years	PIQ (WISC-3)	SM (106.77), CTRL (115.00)	No	0.53
				ANX (105.23), CTRL (115.00)	No	0.63
Manassis et al. (2007b)	ANX (52) ANX + ADHD (35) ADHD (21) CTRL (35)	8–12 years	IQ (WISC-3)	ANX (107.1), CTRL (110.41)	No	0.31
Mogg et al. (2015)	ANX (67) ADHD (67) CTRL (726)	6–12 years	IQ (WISC block design and vocabulary subtests)	ANX (98.6), CTRL (101.5)	No	0.19
Mueller et al. (2015)	ANX (33) CTRL (22)	8–12 years	IQ (Measure not specified)	ANX (109.12), CTRL (115.76)	No	0.48
Rodrigues et al. (2019)	ANX (37) CTRL (34)	7–17 years	IQ (WASI)	ANX (109.56), CTRL (109.94)	No	0.45
Skirbekk et al. (2012)	ANX (41) ADHD (39) ANX + ADHD (25) CTRL (36)	7–13 years	IQ (WASI)	ANX (97.2) < CTRL (109.4)	**Yes**	**0.98**
Toazza et al. (2014)	ANX (16) EXT (11) ANX + EXT (7) CTRL (23)	12–18 years	VIQ (WASI)	ANX (89.1), CTRL (97.1)	No	0.52
			PIQ (WASI)	ANX (86.4), CTRL (95.3)	No	0.62
			FSIQ (WASI)	ANX (86.6), CTRL (95.7)	No	0.66
Toren et al. (2000)	ANX (19) CTRL (14)	6–18 years	FSIQ (WISC-R)	ANX (115.0), CTRL (121.6)	No	0.72
			VIQ (WISC-R)	ANX (109.8), CTRL (117.0)	No	0.66
			PIQ (WISC-R)	ANX (118.9), CTRL (123.7)	No	0.46
Vance et al. (2006)	ANX (25) CTRL (20) ADHD-C (37) DD (37)	6–12 years	FSIQ (WISC-3)	ANX (104.65), CTRL (108.60)	No	0.28
			VIQ (WISC-3)	ANX (107.00), CTRL (111.20)	No	0.33
			PIQ (WISC-3)	ANX (104.67), CTRL (108.60)	No	0.28
Vance et al., 2013	ANX (69) CTRL (116) ADHD (163) ANX + ADHD (116)	6–16 years	FSIQ (WISC-3)	ANX (100.86) < CTRL (110.98)	**Yes**	**0.74**
			VIQ (WISC-3)	ANX (100.94) < CTRL (112.49)	**Yes**	**0.77**
			PIQ (WISC-3)	ANX (102.08) < CTRL (109.15)	**Yes**	**0.48**
Vasa et al. (2007)	ANX (57) CTRL (103)	9–20 years	IQ (K-BIT)	ANX (101.39) < CTRL (105.05)	**Yes**	**0.35**

Studies are presented in alphabetical order. Bold effect sizes indicate a significant difference. Sig Diff = significant difference; IQ = intelligence quotient; FSIQ = Full-scale IQ; VIQ = Verbal IQ; PIQ = Performance IQ

Samples: ADHD = attention-deficit/hyperactivity disorder (i.e., mixed or undefined types); ADHD-C = attention-deficit/hyperactivity disorder – combined; ANX = mixed anxiety; CTRL = healthy control; DD = dysthymic disorder; DEP = depressive disorder; EXT = externalizing disorders; Soc = social anxiety disorder

Measures: K-BIT = Kaufman Brief Intelligence Test; RIST = Reynolds Intellectual Screening Test; WASI = Wechsler Abbreviated Scale of Intelligence; WISC = Wechsler Intelligence Scale for Children

^a^Age range not provided

## Discussion

The present review provides a comprehensive and systematic summary of findings from studies examining differences across multiple domains of cognitive functioning (i.e., episodic memory, language, attention, executive functioning, motor skills, and visual functioning) between youth with anxiety disorders and controls. Two underlying issues emerged. First, in most domains, too few studies were found with consistent results to provide a compelling conclusion. It is possible that this is a result of researchers electing not to, or were unable to, publish null findings. Of note, many of the studies that reported null findings in this review included significant findings for other domains or for non-anxiety groups (e.g., ADHD, depression) that were outside the scope of the present review. However, it is also possible that this is an understudied area. This pattern of limited studies is consistent with reviews in adults (Castaneda et al., 2008; Hedges et al., 2019).

Second, the majority of studies reviewed consisted of small sample sizes (i.e., less than 50 youth per group). Perhaps the association between anxiety disorders and cognitive functioning is present but small and has gone undetected. For studies to be powered to detect a small effect (i.e., Cohen’s d = 0.2; Cohen, 1988), a study would require a sample size of 394 participants per group (assuming a two-tailed test with Power = 0.8 and α = 0.05; Faul et al., 2007). At 50 youth per group and the aforementioned parameters, studies are only powered to detect a moderate effect of d = 0.57. To remedy this for future studies, reviews, or meta-analyses, we calculated and reported effect sizes for all comparisons.

Though strengths in cognitive functioning in anxious youth were not found, several potential weaknesses emerged among domains with multiple studies that allow for the following suggestive conclusions. With regard to attention, visuospatial skills, and one domain of executive functioning (i.e., inhibition), youth with an anxiety disorder did not show differences relative to non-anxious comparison groups. These outcomes are contrary to findings in the adult literature where consistent impairment in executive functioning as well as some findings supporting deficits in attention, visuospatial skills, and memory were found (Castaneda et al., 2008; Hedges et al., 2019). The lack of a difference in attention is rather surprising given the high levels of anxiety and ADHD comorbidity in youth (Jarrett & Ollendick, 2008).

Consistent deficits were found with regard to receptive language as well as fine and gross motor skills. In particular, receptive language skills appear to be worse in youth with SM (but not other anxiety disorders) in contrast to controls, although mean scores were generally within the average range. With this in mind, an explanation and recommendation are provided. Because SM is characterized by anxiety around speaking in certain situations, rather than an inability to speak or comprehend language, this finding may reflect the importance of early language quantity and quality on later language development (Hirsh-Pasek et al., 2015). That is to say that by speaking fewer words, youth with SM are conversing less which may negatively impact their language skills during a critical developmental period. Likewise, parents or siblings of selectively mute youth may accommodate their child’s avoidance by speaking for them and allowing them to converse less (Thompson-Hollands et al., 2014). Thus, it may be important to place a greater emphasis on speaking to youth with SM even if they do not reply, and to similarly encourage them to use educational materials (e.g., phone/tablet applications, television shows) that can help develop receptive language skills. Parents benefit from being mindful of their accommodation (Kagan et al., 2018; Kendall et al., 2020) and, through validation and encouragement of their child’s brave behavior, allow for natural consequences of their child’s avoidance.

Fine and gross motor skills were worse in youth with anxiety disorders compared to controls when measures combined skills, however, when skills were viewed in isolation, variability in outcomes was found. Conversely, motor coordination was not impaired in both studies. It is possible that youth with anxiety experience more motor problems than their non-anxious peers. Youth may be more fearful of social evaluation and, therefore, may engage less in activities that would strengthen their motor skills (e.g., sports). It is also possible that motor problems cause or contribute to anxiety. In fact, some findings suggest that motor problems may be predictive of later anxiety (Piek et al., 2010; Sigurdsson et al., 2002). Given the cross-sectional nature of the included studies, the direction of this relationship is unknown.

Findings were mixed with regard to episodic memory, working memory, and some domains of executive functioning (i.e., planning and cognitive flexibility), with some studies finding deficits in youth with anxiety compared to controls and other studies reporting no differences between the groups. It is possible that the relationship between anxiety and cognitive functioning in youth can fit Yerkes and Dodson’s (1908) classic “Inverted U” model [see Shih and Lin (2017) for a review], where anxiety serves as a strength at some levels and an impediment at others. For example, lower levels of perfectionism in GAD may aid in youth attending to task demands, however, higher levels may prevent youth from thinking flexibily when faced with a challenging assignment. Many studies by design can only test for a linear relationship, yet the relationship may not be linear. This can be partially achieved by examining anxiety dimensionally by severity which was beyond the scope of the present review. As future studies further explore this relationship, it is imperative that non-linear relationship also be considered.

Methodological differences may also be responsible for the mixed findings. Studies used different inclusion criteria, including different diagnostic characterization and different comparison group classification. In fact, the presence of different anxiety disorders has been found to be associated with different cognitive strengths and weaknesses (Micco et al., 2009). For example, the presence of GAD in the diagnostic picture was associated with worse verbal memory while impaired attention was found in youth with SAD (Micco et al., 2009). As most studies aggregated across anxiety disorder, such comparisons were not possible (nor would studies with these small sample sizes be powered to conduct these analyses). Additionally, control group classifications varied based on the study’s goals, with different studies including or excluding participants with subclinical, nonanxiety symptoms. Likewise, the “purity” of anxiety disorder groups varied by study. In one instance, significant differences in initiation between groups disappeared when youth with anxiety plus subthreshold ADHD were excluded from analyses (Korenblum et al., 2007). Additionally, studies used a range of different assessment tools to measure the same cognitive domain. Different measures within the same cognitive domain may also target different aspects of that domain (e.g., immediate vs. delayed memory), contributing to mixed findings. Future studies on specific domains of cognitive functioning should examine specific methodological differences as potential explanations for mixed findings.

Comparisons of IQ support the following conclusion: anxiety disorders in youth are not associated with diminished IQ. In all but one of the 21 studies reporting this comparison, IQ scores for youth with anxiety disorders fell within the average range. This finding is unsurprising because, although anxiety has been found to negatively impact test performance (von der Embse & Hasson, 2012), there is no theoretical basis to hypothesize a difference. Only test anxiety – but not state anxiety, trait anxiety, or general anxiety – was significantly associated with lower performance IQ in college students (Hopko et al., 2005). Neuropsychologists and others assessing youth with anxiety should be observant of behaviors indicative of test anxiety and contextualize any findings of lower IQ within those behavioral observations and the present review’s findings of average IQ for youth with anxiety.

## Future Directions

Due to the few studies in each domain, further research is needed to truly examine any potential strengths or weaknesses in cognitive functioning in youth with anxiety disorders. These studies should include larger samples that are representative of the population to increase the generalizability of findings. Above and beyond additional exploration of these domains, three important avenues of future research warrant discussion including (a) the role of cognitive functioning as a predictor of anxiety treatment outcome (b) the effect of treatment on cognitive functioning, and (c) the course and specificity of anxiety and potential impairment in cognitive functioning.

## Cognitive Functioning as a Treatment Predictor

When considering the treatment implications of the present review’s findings, an area of strength or weakness typically found in youth with anxiety, may differentially predict treatment outcome for youth with either greater or lesser ability. No strengths emerged, however, two weaknesses were identified, receptive language and motor skills. In youth with SM, a decrease in anxiety around speaking progressing to an increased use of various forms of language is a treatment goal (Furr et al., 2020). Thus, youth with impaired receptive language may fare worse in treatment if receptive language difficulties impede their ability to converse. Future research should evaluate receptive language as a predictor of treatment outcome. Likewise, motor skills warrant future research as a predictor. As motor problems may be predictive of later anxiety (Piek et al., 2010; Sigurdsson et al., 2002), motor problems, either in the form of a history of developmental motor delay or present impairment, may interact with anxiety and compound the nature of its interference. Given that play is an important element of youths’ lives (Yogman et al., 2018), youth with worse motor skills may be fearful of their abilities and performance or of potential social evaluation. Thus, these youth may be more reticent to engage in exposure tasks which would hinder progress. Finally, as more research is conducted and conclusions are able to be drawn, domains where findings were presently mixed (i.e., episodic memory, working memory, planning, and cognitive flexibility) as well as domains where too few studies were conducted to allow for characterization, may provide avenues for future research on predictors of treatment outcome.

## Effect of Treatment on Cognitive Functioning

CBT is effective in reducing both the symptoms of anxiety and the long-term functional consequences of anxiety (Ginsburg et al., 2011, 2014; Swan et al., 2018). However, although findings indicate changes in cognitive content (e.g., self-talk; Kendall & Treadwell, 2007; Kendall et al., 2016), less is known about the effect of CBT on cognitive functioning in youth. If the impairment in cognitive functioning is associated with anxiety rather than concurrent with anxiety, treatment of anxiety may improve performance. In youth with both ADHD and anxiety, treatment of anxiety was found to improve inhibition, cognitive flexibility, and inattentive and hyperactive behaviors, though treatment did not impact working memory or attention deficits (Denis et al., 2016). In another study, increased inhibitory control and reduced attentional bias to threat were found in youth with anxiety following CBT (Hadwin & Richards, 2016). It is possible that CBT may strengthen certain domains above and beyond the levels reviewed here. Further research is needed to examine the effect of therapy for anxiety on cognitive functioning across a range of domains. Additionally, given the prevalence of medication prescription for anxiety in youth (Whiteside et al., 2019), the effect of medication for anxiety on cognitive performance should also be studied. An examination of the side effects of a 6-week course of sertraline, a selective serotonin reuptake inhibitors commonly used to treat anxiety in youth (Bushnell et al., 2018), indicated that the medication does not impact attention or inhibition, but did impact susceptibility to interference on a verbal memory task (Günther et al., 2005). Though promising that minimal side effects were found, youth are typically prescribed medication for longer than six weeks. Future research should assess cognitive functioning at the time of medication termination as anxiety will likely be reduced and the true impact of medication on cognitive performance associated with anxiety can be assessed.

Related, CBT is often adapted when youth present with intellectual or developmental disabilities, with a greater focus placed on behavioral components rather than cognitive ones (Hronis et al., 2017). Domains with mixed findings (e.g., visual memory and working memory) may reflect variability within youth with anxiety, indicative of an individual effect rather than an overall effect and necessitating a personalized approach. For instance, youth presenting with working memory weaknesses may benefit from a working memory training to supplement traditional CBT. One such training has been found to reduce anxiety symptoms and attentional bias to threat while also improving working memory and inhibitory control (Hadwin & Richards, 2016). Conversely, if problem solving is an issue, time spent working on that technique may be better spent on a different one. These cognitive functioning-related adaptations may be a similar avenue to personalize treatment for all youth with anxiety and increase response rates from about 60% (Kendall et al., 2008; Walkup et al., 2008).

## Course and Specificity of Anxiety and Cognitive Functioning

The course of anxiety and potential impairment in cognitive functioning largely remains uncertain. Does anxiety precede the deficits in cognitive functioning, do the deficits precede anxiety, or do both onset at the same time? As noted previously, motor problems may be predictive of later anxiety (Piek et al., 2010; Sigurdsson et al., 2002), however, less is known about other domains of cognitive functioning. Longitudinal studies are needed to probe this relationship. Furthermore, as most studies collapsed across anxiety disorders, it remains unclear whether any strengths or weaknesses (with the exception of receptive language in youth with SM) are exclusive to certain anxiety disorders. Understanding the course and specificity of this potential relationship will have implications on the prevention and treatment of anxiety disorders in youth and also may provide insight into the etiology of anxiety disorders.

## Conclusion

The present review highlights that research on cognitive functioning in youth with an anxiety disorder remains in its infancy. Questions remain about strengths and weaknesses, treatment effects, and the course and specificity of the relationship between anxiety and cognitive functioning. As anxiety disorders are often theorized as disorders of cognition, consideration of how cognitive functioning and anxiety interact is important. Future research may require shifting conceptualizations from linear relationships to more complex models that incorporate changes in relationship by degree of impairment.

## References

[CR1] American Psychiatric Association (2013). Diagnostic and statistical manual of mental disorders.

[CR2] Baving L, Rellum T, Laucht M, Schmidt MH (2004). Attentional enhancement to NoGo stimuli in anxious children. Journal of Neural Transmission.

[CR3] Benton Sivan, A. (1991). *Benton visual retention test*, 5th edn. 10.1037/t14985-000

[CR4] Bushnell GA, Compton SN, Dusetzina SB, Gaynes BN, Brookhart MA, Walkup JT, Rynn MA, Stürmer T (2018). Treating pediatric anxiety: Initial use of SSRIs and other anti-anxiety prescription medications. The Journal of Clinical Psychiatry.

[CR5] Cardinale EM, Subar AR, Brotman MA, Leibenluft E, Kircanski K, Pine DS (2019). Inhibitory control and emotion dysregulation: A framework for research on anxiety. Development and Psychopathology.

[CR6] Castaneda AE, Tuulio-Henriksson A, Marttunen M, Suvisaari J, Lönnqvist J (2008). A review on cognitive impairments in depressive and anxiety disorders with a focus on young adults. Journal of Affective Disorders.

[CR7] Cohen J (1988). Statistical power analysis for the behavioral sciences.

[CR8] Costello EJ, Mustillo S, Erkanli A, Keeler G, Angold A (2003). Prevalence and development of psychiatric disorders in childhood and adolescence. Archives of General Psychiatry.

[CR9] Cowie J, Clementi MA, Alfano CA (2018). Examination of the intolerance of uncertainty construct in youth with generalized anxiety disorder. Journal of Clinical Child & Adolescent Psychology.

[CR10] Craske MG, Kircanski K, Zelikowsky M, Mystkowski J, Chowdhury N, Baker A (2008). Optimizing inhibitory learning during exposure therapy. Behaviour Research and Therapy.

[CR11] Davis TE, Ollendick TH, Nebel-Schwalm M (2008). Intellectual ability and achievement in anxiety-disordered children: A clarification and extension of the literature. Journal of Psychopathology and Behavioral Assessment.

[CR12] Denis I, Guay M-C, Foldes-Busque G, BenAmor L (2016). Effect of treating anxiety disorders on cognitive deficits and behaviors associated with attention deficit hyperactivity disorder: A preliminary study. Child Psychiatry & Human Development.

[CR13] Dooley B, Fitzgerald A, Giollabhui NM (2015). The risk and protective factors associated with depression and anxiety in a national sample of Irish adolescents. Irish Journal of Psychological Medicine.

[CR14] Dudeney J, Sharpe L, Hunt C (2015). Attentional bias towards threatening stimuli in children with anxiety: A meta-analysis. Clinical Psychology Review.

[CR15] Ekornås B, Lundervold AJ, Tjus T, Heimann M (2010). Anxiety disorders in 8–11-year-old children: Motor skill performance and self-perception of competence. Scandinavian Journal of Psychology.

[CR16] Emerson C, Mollet G, Harrison D (2005). Anxious-depression in boys: An evaluation of executive functioning. Archives of Clinical Neuropsychology.

[CR17] Essau CA, Lewinsohn PM, Olaya B, Seeley JR (2014). Anxiety disorders in adolescents and psychosocial outcomes at age 30. Journal of Affective Disorders.

[CR18] Faul F, Erdfelder E, Lang A-G, Buchner A (2007). G*Power 3: A flexible statistical power analysis program for the social, behavioral, and biomedical sciences. Behavior Research Methods.

[CR19] Furr, J. M., Sanchez, A. L., Hong, N., & Comer, J. S. (2020). Chapter 6—Exposure therapy for childhood selective mutism: Principles, practices, and procedures. In T. S. Peris, E. A. Storch, & J. F. McGuire (Eds.), *Exposure therapy for children with anxiety and OCD* (pp. 113–142). Academic Press. 10.1016/B978-0-12-815915-6.00006-8

[CR20] Ginsburg GS, Becker EM, Keeton CP, Sakolsky D, Piacentini J, Albano AM, Compton SN, Iyengar S, Sullivan K, Caporino N, Peris T, Birmaher B, Rynn M, March J, Kendall PC (2014). Naturalistic follow-up of youths treated for pediatric anxiety disorders. JAMA Psychiatry.

[CR21] Ginsburg GS, Sakolsky D, Piacentini J, Walkup JT, Coffey KA, Keeton CP, Iyengar S, Kendall PC, Compton SN, Albano AM, Sherrill J, Rynn MA, McCracken JT, Bergman L, Birmaher B, March J (2011). Remission after acute treatment in children and adolescents with anxiety disorders: Findings from the CAMS. Journal of Consulting and Clinical Psychology.

[CR22] Goodall J, Fisher C, Hetrick S, Phillips L, Parrish EM, Allott K (2018). Neurocognitive functioning in depressed young people: A systematic review and meta-analysis. Neuropsychology Review.

[CR23] Gosch EA, Flannery-Schroeder E, Mauro CF, Compton SN (2006). Principles of cognitive-behavioral therapy for anxiety disorders in children. Journal of Cognitive Psychotherapy.

[CR24] Günther T, Holtkamp K, Jolles J, Herpertz-Dahlmann B, Konrad K (2004). Verbal memory and aspects of attentional control in children and adolescents with anxiety disorders or depressive disorders. Journal of Affective Disorders.

[CR25] Günther T, Holtkamp K, Jolles J, Herpertz-Dahlmann B, Konrad K (2005). The influence of sertraline on attention and verbal memory in children and adolescents with anxiety disorders. Journal of Child and Adolescent Psychopharmacology.

[CR26] Hadwin JA, Richards HJ (2016). Working memory training and CBT reduces anxiety symptoms and attentional biases to threat: A preliminary study. Frontiers in Psychology.

[CR27] Hardin MG, Schroth E, Pine DS, Ernst M (2007). Incentive-related modulation of cognitive control in healthy, anxious, and depressed adolescents: Development and psychopathology related differences. Journal of Child Psychology and Psychiatry.

[CR28] Hedges, D., Farrer, T. J., Bigler, E. D., & Hopkins, R. O. (2019). Cognition in anxiety disorders. In D. Hedges, T. J. Farrer, E. D. Bigler, & R. O. Hopkins (Eds.), *The brain at risk: Associations between disease and cognition* (pp. 37–48). Springer. 10.1007/978-3-030-14260-5_3

[CR29] Henderson, S. E., Sugden, D., & Barnett, A. L. (2007). *Movement assessment battery for children-2*. 10.1037/t55281-000

[CR30] Higa-McMillan CK, Francis SE, Rith-Najarian L, Chorpita BF (2016). Evidence base update: 50 years of research on treatment for child and adolescent anxiety. Journal of Clinical Child & Adolescent Psychology.

[CR31] Hirsh-Pasek K, Adamson LB, Bakeman R, Owen MT, Golinkoff RM, Pace A, Yust PKS, Suma K (2015). The contribution of early communication quality to low-Income children’s language success. Psychological Science.

[CR32] Holder LJ, Prasad A, Han J, Torok M, Wong QJJ (2021). Shifting as a key executive function underlying cognitive restructuring for individuals with elevated social anxiety. Psychology and Psychotherapy: Theory, Research and Practice.

[CR33] Hopko DR, Crittendon JA, Grant E, Wilson SA (2005). The impact of anxiety on performance IQ. Anxiety, Stress & Coping.

[CR34] Hronis A, Roberts L, Kneebone II (2017). A review of cognitive impairments in children with intellectual disabilities: Implications for cognitive behaviour therapy. British Journal of Clinical Psychology.

[CR35] Humes GE, Welsh MC, Retzlaff P, Cookson N (1997). Towers of Hanoi and London: Reliability and validity of two executive function tasks. Assessment.

[CR36] Hybel KA, Mortensen EL, Lambek R, Thastum M, Thomsen PH (2017). Cool and hot aspects of executive function in childhood obsessive-compulsive disorder. Journal of Abnormal Child Psychology.

[CR37] Ingram RE, Kendall PC (1987). The cognitive side of anxiety. Cognitive Therapy and Research.

[CR38] Jarrett MA, Ollendick TH (2008). A conceptual review of the comorbidity of attention-deficit/hyperactivity disorder and anxiety: Implications for future research and practice. Clinical Psychology Review.

[CR39] Jarros RB, Salum GA, da Silva CTB, Toazza R, Becker N, Agranonik M, de Salles JF, Manfro GG (2017). Attention, memory, visuoconstructive, and executive task performance in adolescents with anxiety disorders: A case-control community study. Trends in Psychiatry and Psychotherapy.

[CR40] Jazbec S, McClure E, Hardin M, Pine DS, Ernst M (2005). Cognitive control under contingencies in anxious and depressed adolescents: An Anti-saccade task. Biological Psychiatry.

[CR41] John, S. C. F. (2005). *Distinguishing anxiety in childhood: Clinical and cognitive characteristics* [Doctoral dissertation, University of Toronto]. ProQuest Dissertations Publishing.

[CR42] Johnco C, Wuthrich VM, Rapee RM (2013). The role of cognitive flexibility in cognitive restructuring skill acquisition among older adults. Journal of Anxiety Disorders.

[CR43] Johnson, D. A., Roethig-Johnston, K., & Middleton, J. (1988). Development and evaluation of an attentional test for head injured children—1: Information processing capacity in a normal sample. *Journal of Child Psychology and Psychiatry*, *29*(2), 199–208. 10.1111/j.1469-7610.1988.tb00704.x 10.1111/j.1469-7610.1988.tb00704.x 3372616

[CR44] Kagan ER, Frank HE, Kendall PC (2018). Accommodation in youths’ mental health: Evidence and issues. Current Directions in Psychological Science.

[CR45] Kakebeeke TH, Jongmans MJ, Dubowitz LMS, Schoemaker MM, Henderson SM (1993). Some aspects of the reliability of Touwen’s examination of the child with minor neurological dysfunction. Developmental Medicine & Child Neurology.

[CR46] Kendall PC, Cummings CM, Villabø MA, Narayanan MK, Treadwell K, Birmaher B, Compton S, Piacentini J, Sherrill J, Walkup J, Gosch E, Keeton C, Ginsburg G, Suveg C, Albano AM (2016). Mediators of change in the child/adolescent anxiety multimodal treatment study. Journal of Consulting and Clinical Psychology.

[CR47] Kendall PC, Hudson JL, Gosch E, Flannery-Schroeder E, Suveg C (2008). Cognitive-behavioral therapy for anxiety disordered youth: A randomized clinical trial evaluating child and family modalities. Journal of Consulting and Clinical Psychology.

[CR48] Kendall PC, Norris LA, Rabner JC, Crane ME, Rifkin LS (2020). Intolerance of uncertainty and parental accommodation: Promising targets for personalized intervention for youth anxiety. Current Psychiatry Reports.

[CR49] Kendall PC, Treadwell KRH (2007). The role of self-statements as a mediator in treatment for youth with anxiety disorders. Journal of Consulting and Clinical Psychology.

[CR50] Kim KL, Christensen RE, Ruggieri A, Schettini E, Freeman JB, Garcia AM, Flessner C, Stewart E, Conelea C, Dickstein DP (2019). Cognitive performance of youth with primary generalized anxiety disorder versus primary obsessive–compulsive disorder. Depression and Anxiety.

[CR51] Korenblum CB, Chen SX, Manassis K, Schachar RJ (2007). Performance monitoring and response inhibition in anxiety disorders with and without comorbid ADHD. Depression and Anxiety.

[CR52] Kristensen H, Oerbeck B (2006). Is selective mutism associated with deficits in memory span and visual memory? An exploratory case–control study. Depression and Anxiety.

[CR53] Kristensen H, Torgersen S (2008). Is social anxiety disorder in childhood associated with developmental deficit/delay?. European Child & Adolescent Psychiatry.

[CR54] Latinjak AT, Morin A, Brinthaupt TM, Hardy J, Hatzigeorgiadis A, Kendall PC, Neck C, Oliver EJ, Puchalska-Wasyl MM, Tovares AV, Winsler A (2023). Self-talk: An interdisciplinary review and transdisciplinary model. Review of General Psychology.

[CR55] Lubow RE, Toren P, Laor N, Kaplan O (2000). The effects of target and distractor familiarity on visual search in anxious children: Latent inhibition and novel pop-out. Journal of Anxiety Disorders.

[CR56] Manassis K, Tannock R, Barbosa J (2000). Dichotic listening and response inhibition in children with comorbid anxiety disorders and ADHD. Journal of the American Academy of Child & Adolescent Psychiatry.

[CR57] Manassis K, Tannock R, Garland EJ, Minde K, McInnes A, Clark S (2007). The sounds of silence: Language, cognition, and anxiety in selective mutism. Journal of the American Academy of Child & Adolescent Psychiatry.

[CR58] Manassis K, Tannock R, Young A, Francis-John S (2007). Cognition in anxious children with attention deficit hyperactivity disorder: A comparison with clinical and normal children. Behavioral and Brain Functions.

[CR59] Mason, L. S. H. (2017). *Misunderstanding of nonverbal communication in anxious and nonclinical youth* [Doctoral dissertation, Fairleigh Dickinson University]. ProQuest Dissertations Publishing.

[CR60] Merikangas KR, He J, Burstein M, Swanson SA, Avenevoli S, Cui L, Benjet C, Georgiades K, Swendsen J (2010). Lifetime prevalence of mental disorders in U.S. adolescents: Results from the National Comorbidity Survey Replication-Adolescent Supplement (NCS-A). Journal of the American Academy of Child & Adolescent Psychiatry.

[CR61] Micco JA, Henin A, Biederman J, Rosenbaum JF, Petty C, Rindlaub LA, Murphy M, Hirshfeld-Becker DR (2009). Executive functioning in offspring at risk for depression and anxiety. Depression and Anxiety.

[CR62] Milic MI, Carl T, Rapee RM (2020). Similarities and differences between young children with selective mutism and social anxiety disorder. Behaviour Research and Therapy.

[CR63] Mogg K, Salum GA, Bradley BP, Gadelha A, Pan P, Alvarenga P, Rohde LA, Pine DS, Manfro GG (2015). Attention network functioning in children with anxiety disorders, attention-deficit/hyperactivity disorder and non-clinical anxiety. Psychological Medicine.

[CR64] Mueller SC, Shechner T, Rosen D, Nelson EE, Pine DS, Ernst M (2015). Incidental threat during visuospatial working memory in adolescent anxiety: An emotional memory-guided saccade task. Depression and Anxiety.

[CR65] Nowakowski ME, Cunningham CE, McHolm AE, Evans MA, Edison S, Pierre JS, Boyle MH, Schmidt LA (2009). Language and academic abilities in children with selective mutism. Infant and Child Development.

[CR66] Piek JP, Barrett NC, Smith LM, Rigoli D, Gasson N (2010). Do motor skills in infancy and early childhood predict anxious and depressive symptomatology at school age?. Human Movement Science.

[CR67] Reitan, R. M., & Wolfson, D. (1993). *The Halstead-Reitan neuropsychological test battery: Theory and clinical interpretation*, 2nd edn. Neurospychology Press. Retrieved from https://www.pearsonassessments.com/store/usassessments/en/Store/Professional-Assessments/Cognition-%26-Neuro/Wechsler-Intelligence-Scale-for-Children-%7C-Fourth-Edition/p/100000310.html

[CR68] Rodrigues CL, de Rocca CCA, Serafim A, dos Santos B, Asbahr FR (2019). Impairment in planning tasks of children and adolescents with anxiety disorders. Psychiatry Research.

[CR69] Schniering CA, Rapee RM (2002). Development and validation of a measure of children’s automatic thoughts: The children’s automatic thoughts scale. Behaviour Research and Therapy.

[CR70] Schniering CA, Rapee RM (2004). The structure of negative self-statements in children and adolescents: A confirmatory factor-analytic approach. Journal of Abnormal Child Psychology.

[CR71] Seeley JR, Kosty DB, Farmer RF, Lewinsohn PM (2011). The modeling of internalizing disorders based on patterns of lifetime comorbidity: Associations with psychosocial functioning and psychiatric disorders among first–degree relatives. Journal of Abnormal Psychology.

[CR72] Sheslow, D., & Adams, W. (1990). *Wide range assessment of memory and learning*. Jastak. Retrieved from https://www.pearsonassessments.com/store/usassessments/en/Store/Professional-Assessments/Cognition-%26-Neuro/Wechsler-Intelligence-Scale-for-Children-%7C-Fourth-Edition/p/100000310.html

[CR73] Shih H-H, Lin M-J (2017). Does anxiety affect adolescent academic performance? The inverted-U hypothesis revisited. Journal of Labor Research.

[CR74] Sigurdsson E, van Os J, Fombonne E (2002). Are impaired childhood motor skills a risk factor for adolescent anxiety? Results from the 1958 U.K. Birth Cohort and the National Child Development Study. American Journal of Psychiatry.

[CR75] Skirbekk B, Hansen BH, Oerbeck B, Wentzel-Larsen T, Kristensen H (2012). Motor impairment in children with anxiety disorders. Psychiatry Research.

[CR76] Swan AJ, Kendall PC (2016). Fear and missing out: Youth anxiety and functional outcomes. Clinical Psychology: Science and Practice.

[CR77] Swan AJ, Kendall PC, Olino T, Ginsburg G, Keeton C, Compton S, Piacentini J, Peris T, Sakolsky D, Birmaher B, Albano AM (2018). Results from the Child/Adolescent Anxiety Extended Long-term Study (CAMELS): Functional outcomes. Journal of Consulting and Clinical Psychology.

[CR78] Thompson-Hollands J, Kerns CE, Pincus DB, Comer JS (2014). Parental accommodation of child anxiety and related symptoms: Range, impact, and correlates. Journal of Anxiety Disorders.

[CR79] Toazza R, Salum GA, Flores SM, Jarros RB, Pine DS, de Salles JF, Manfro GG (2014). Phonemic verbal fluency is associated with pediatric anxiety disorders: Evidence from a community study. Journal of Child and Adolescent Psychopharmacology.

[CR80] Toren P, Sadeh M, Wolmer L, Eldar S, Koren S, Weizman R, Laor N (2000). Neurocognitive correlates of anxiety disorders in children: A preliminary report. Journal of Anxiety Disorders.

[CR81] Treadwell KRH, Kendall PC (1996). Self-talk in youth with anxiety disorders: States of mind, content specificity, and treatment outcome. Journal of Consulting and Clinical Psychology.

[CR82] Van Ameringen M, Mancini C, Farvolden P (2003). The impact of anxiety disorders on educational achievement. Journal of Anxiety Disorders.

[CR83] Vance A, Arduca Y, Sanders M, Karamitsios M, Hall N, Hetrick S (2006). Attention deficit hyperactivity disorder, combined type, dysthymic disorder and anxiety disorders: Differential patterns of neurodevelopmental deficits. Psychiatry Research.

[CR84] Vance A, Ferrin M, Winther J, Gomez R (2013). Examination of spatial working memory performance in children and adolescents with attention deficit hyperactivity disorder, combined type (ADHD-CT) and anxiety. Journal of Abnormal Child Psychology.

[CR85] Vasa RA, Roberson-Nay R, Klein RG, Mannuzza S, Moulton JL, Guardino M, Merikangas A, Carlino AR, Pine DS (2007). Memory deficits in children with and at risk for anxiety disorders. Depression and Anxiety.

[CR86] von der Embse N, Hasson R (2012). Test anxiety and high-stakes test performance between school settings: Implications for educators. Preventing School Failure: Alternative Education for Children and Youth.

[CR87] Walkup JT, Albano AM, Piacentini J, Birmaher B, Compton SN, Sherrill JT, Ginsburg GS, Rynn MA, McCracken J, Waslick B, Iyengar S, March JS, Kendall PC (2008). Cognitive behavioral therapy, sertraline, or a combination in childhood anxiety. New England Journal of Medicine.

[CR88] Walter HJ, Bukstein OG, Abright AR, Keable H, Ramtekkar U, Ripperger-Suhler J, Rockhill C (2020). Clinical practice guideline for the assessment and treatment of children and adolescents with anxiety disorders. Journal of the American Academy of Child & Adolescent Psychiatry.

[CR89] Wechsler, D. (1991). *Wechsler intelligence scale for children*, 3rd edn. Psychological Corporation. Retrieved from https://www.pearsonassessments.com/store/usassessments/en/Store/Professional-Assessments/Cognition-%26-Neuro/Wechsler-Intelligence-Scale-for-Children-%7C-Fourth-Edition/p/100000310.html

[CR90] Wechsler, D. (2003). *Wechsler Intelligence Scale for Children*, 4th edn. Psychological Corporation. Retrieved from https://www.pearsonassessments.com/store/usassessments/en/Store/Professional-Assessments/Cognition-%26-Neuro/Wechsler-Intelligence-Scale-for-Children-%7C-Fourth-Edition/p/100000310.html

[CR91] Werry JS, Elkind GS, Reeves JC (1987). Attention deficit, conduct, oppositional, and anxiety disorders in children: III. Laboratory differences. Journal of Abnormal Child Psychology.

[CR92] Whiteside SP, Sim LA, Olsen MW, Hord MK (2019). The five-year course of medication treatment in childhood anxiety disorders. The Journal of Clinical Psychiatry.

[CR93] Woodward LJ, Fergusson DM (2001). Life course outcomes of young people with anxiety disorders in adolescence. Journal of the American Academy of Child & Adolescent Psychiatry.

[CR94] Yerkes RM, Dodson JD (1908). The relation of strength of stimulus to rapidity of habit formation. Journal of Comparative Neurology & Psychology.

[CR95] Yogman, M., Garner, A., Hutchinson, J., Hirsh-Pasek, K., Golinkoff, R. M., Health, C. on P. A. of C. and F., Media, C. on C. A., Baum, R., Gambon, T., Lavin, A., Mattson, G., Wissow, L., Hill, D. L., Ameenuddin, N., Chassiakos, Y. (Linda) R., Cross, C., Boyd, R., Mendelson, R., Moreno, M. A., … Smith, J. (2018). The power of play: A pediatric role in enhancing development in young children. *Pediatrics*, *142*(3), e20182058. 10.1542/peds.2018-2058 10.1542/peds.2018-2058 30126932

